# Psychophysical Laws and the Superorganism

**DOI:** 10.1038/s41598-018-22616-y

**Published:** 2018-03-12

**Authors:** Andreagiovanni Reina, Thomas Bose, Vito Trianni, James A. R. Marshall

**Affiliations:** 1 Department of Computer Science, University of Sheffield, Sheffield, S1 4DP UK; 2 ISTC, Italian National Research Council, Rome, Italy

## Abstract

Through theoretical analysis, we show how a superorganism may react to stimulus variations according to psychophysical laws observed in humans and other animals. We investigate an empirically-motivated honeybee house-hunting model, which describes a value-sensitive decision process over potential nest-sites, at the level of the colony. In this study, we show how colony decision time increases with the number of available nests, in agreement with the Hick-Hyman law of psychophysics, and decreases with mean nest quality, in agreement with Piéron’s law. We also show that colony error rate depends on mean nest quality, and difference in quality, in agreement with Weber’s law. Psychophysical laws, particularly Weber’s law, have been found in diverse species, including unicellular organisms. Our theoretical results predict that superorganisms may also exhibit such behaviour, suggesting that these laws arise from fundamental mechanisms of information processing and decision-making. Finally, we propose a combined psychophysical law which unifies Hick-Hyman’s law and Piéron’s law, traditionally studied independently; this unified law makes predictions that can be empirically tested.

## Introduction

Psychophysics, introduced in the nineteenth century by Fechner^1,2^, studies the relationship between stimulus intensity and its perception in the human brain. This relationship has been explained through a set of psychophysical laws that hold in a wide spectrum of sensory domains, such as sound loudness, musical pitch, image brightness, time duration, vibrotactile frequency, weight, and numerosity^1–8^. More recently, numerous studies have shown that a wide range of organisms at various levels of complexity obey these laws. For instance, Weber’s law^1,2^, which Fechner named after his mentor Weber, holds in humans as well as in other mammals^9^, fish^10^, birds^11^ and insects^12,13^. Surprisingly, also organisms without a brain can display such behaviour, for instance slime moulds^14^ and other unicellular organisms^15,16^.

In this study, for the first time, we show that superorganismal behaviour, such as honeybee nest-site selection, may obey the same psychophysical laws displayed by humans in sensory discriminatory tasks. Weber’s law has been acknowledged in the behaviour of individual insects^12,13^, however, it has not successfully been investigated at the colony-level, considered as a single superorganism. In our previous work^17,18^, through stability analysis (see Sec.Weber’s Law) of deterministic models, we found colony-level regime changes that could possibly lead to dynamics in agreement with Weber’s law. In this study, through a thorough analysis and stochastic computational simulations, we more fully investigate the adherence to three psychophysical laws: Weber’s, Hick-Hyman’s, and Piéron’s law, which relate decision conditions to decision time and accuracy.

**Figure 1:**
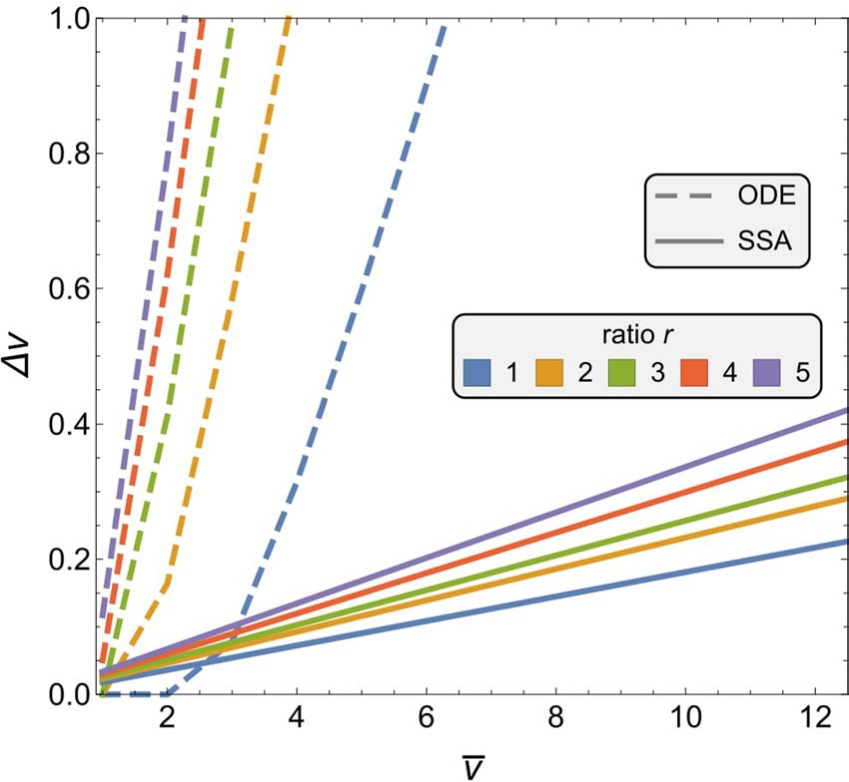
Comparison between analytical results from the ODE system of Eqs (1) and (2) and the computational results from the SSA with system size *S* = 500, see Sec. 5.1. The analytical lines show the bifurcation from bistability (left side, where noise may lead to inferior option) to a single-attractor phase (right side, where convergence to the superior option is guaranteed)^18^. The SSA results display the minimum difference for which the model correctly discriminates; this happens in the bistable regime. The SSA lines are fitted through linear regression (Supplementary Table S1).

Finding psychophysical laws in a non-neurological model contributes to the identification of general mechanisms generating these patterns and the adaptive benefits that led to them^19^. Our findings are based on a model derived from previous empirical observations of house-hunting honeybees^20^. However, a model response in agreement with psychophysical laws does not guarantee that the same response would be found in social insects. However, we believe our contribution is a valuable testable prediction; this may motivate empirical collective behaviour researchers to confirm that a superorganism—not limited to honeybees—can display cognitive abilities with characteristics comparable to higher order organisms^21–23^. Our study can also be of interest to psychologists because it proposes an amalgamated psychophysical law that subsumes the traditionally separate Hick-Hyman’s and Piéron’s laws.

## Model

In spring, colonies of the European honey bee (*Apis mellifera*) reproduce though fission, and the resident queen leaves the nest together with thousands of scout bees. Part of the swarm protects the queen while the other part of the swarm makes a decision on the best available nesting location. Scout bees explore the surrounding environment and, having located a potential nest-site, return to the swarm to actively recruit other scouts to that site, through the waggle dance. When a scout committed to one nest-site encounters a scout dancing for another, it may deliver a stop-signal; a bee that receives several stop-signals reverts to an uncommitted state. The colony makes a decision when the honeybees committed to the same option reach a quorum *Q*^24^. In our study psychophysical laws are measured at the colony level and the organism’s response to varying stimulus strengths corresponds to the colony’s response to varying nest-site qualities.

This nest-site selection process as been modelled^18,20^ as:

1 $$\begin{array}{rcl}\frac{d{x}_{i}}{dt} & = & {\gamma }_{i}\,{x}_{u}-{\alpha }_{i}\,{x}_{i}+{\rho }_{i}\,{x}_{u}\,{x}_{i}-\sum _{j=1}^{n}\,{x}_{j}\,{\beta }_{ji}\,{x}_{i},\quad i\in \{1,\ldots ,n\},\\ {x}_{u} & = & 1-\sum _{i=1}^{n}\,{x}_{i}\end{array}$$

This model describes the changes of *x*_*i*_ over time, which represent the proportion of bees committed to nest *i* with *i* ∈ {1, …, *n*}, with *x*_*u*_ representing the proportion of uncommitted bees. The variation of committment to nest-site *i* is determined by four behaviours: (i) increase at rate *γ*_*i*_ through individual discovery, (ii) decrease at rate *α*_*i*_ through individual abandonment, (iii) increase at rate *x*_*u*_*ρ*_*i*_ through recruitment upon interaction (in the form of waggle dance), and (iv) decrease at rate *x*_*j*_*β*_*ji*_ (with *i* ≠ *j*) through cross-inhibition upon interaction (in the form of stop signals).

When a scout bee visits a potential nest-site *i*, she estimates its quality *v*_*i*_ and modulates her behaviour accordingly, showing higher activity in support of better quality nest-sites^25^. Owing on these observations, in a previous study^18^ we proposed a value sensitive parameterisation:

2 $${\gamma }_{i}=k\,{v}_{i},\quad {\alpha }_{i}=k\,{v}_{i}^{-1},\quad {\rho }_{i}=h\,{v}_{i},\quad {\beta }_{ij}=h\,{v}_{i},$$

where the parameters *k* and *h* modulate the strength of individual and signalling behaviours in response to perceived site quality, respectively. Equation (2) generalises the parameterisation proposed in^17^ to allow effective choices in best-of-n decision problems. Empirical observations^20,25^ motivate such a parameterisation and show that individual recruitment behaviour is proportional to estimated nest quality. As in^18^, we reduce the analysis to the single control parameter *r* = *h*/*k* which is the ratio between signalling and individual behaviour. When $r\gg 1$, the decision dynamics are strongly influenced by recruitment and cross-inhibition. In this case, the first few discovered options can quickly gain momentum and are very likely to be selected, leading to quick decisions with low accuracy. Instead, when *r* < 1 interactions among bees are less relevant and the dynamics are dominated by individual discovery and abandonment. In this case, reaching a quorum is not guaranteed, especially when options have similar qualities (*v*_*i*_), as shown in^18^. In the special case of *r* = 0, bees spread among the available options quasi-proportionally with respect to the options’ quality.

## Psychophysical Laws

We analysed the dynamics of the decision model of Eq. (1) under the parameterisation (2) by introducing random fluctuations caused by the finite size of the system (*i*.*e*. the system is composed of a finite number of bees *S*). The model of Eq. (1) can be described in the form of a master equation, as done in^20,26^, which allows the analysis of the finite-size system dynamics. We approximated the solution of the master equation through the *stochastic simulation algorithm* (SSA)^27^.

### Weber’s Law

Weber’s law^1,2^ states that the minimum difference between two stimuli Δ*v* (also known as *just noticeable difference*) that an organism can correctly discriminate is a constant fraction *w* of the base stimulus strength:

3 $$w=\frac{{\rm{\Delta }}v}{\bar{v}};$$

in our analysis, $\bar{v}$ represents the mean stimulus strength (*i*.*e*., the mean nest-site quality).

Figure 1 shows a comparison between the results of the SSA and the previously proposed bifurcation analysis^17,18^, which was based only on the phase transition between bistable and single-attractor regimes. Analogous to these previous results, we find a linear relationship between the mean quality $\bar{v}$ and the just noticeable difference (Δ*v*), however, with a different slope. Indeed the two lines represent two different measures. The phase transition (dashed lines) shows a change of regime in the mean-field system, passing from a phase with two attractors (representing possible decisions for either option) to a phase with a single attractor in favour to the superior option only. This change of regime hinted at the possibility of agreement with Weber’s law^17,18^, which however had not yet been shown. In contrast, in this study, through SSA simulations we precisely determine the actual decision outcomes (*i*.*e*. when one committed population surpasses the quorum *Q*) and compute the Weber fraction *w* accordingly (displayed as solid lines in Fig. 1, see Sec. 5 for details on the SSA results). Figure 1 show that the model can correctly discriminate between options within the bistability regime. In this regime, having an attractor for each option introduces the possibility of converging to the inferior option due to random fluctuations, although, when the quality difference is large enough (${\rm{\Delta }}v\ge w\bar{v}$), the swarm is able to select the highest value option.

Figure 1 also shows how the discriminability of signals varies as a function of the signalling ratio *r*; that is, the Weber fraction *w* grows with *r*. Increasing levels of signalling reduce the ability of the organism to discriminate between similar stimuli. The adaptive pressure to keep low levels of signalling must be balanced with the pressure to increase signalling in order to speed up the decision process (Sec. 3.2) and to break decision deadlocks (see^18^).

**Figure 2:**
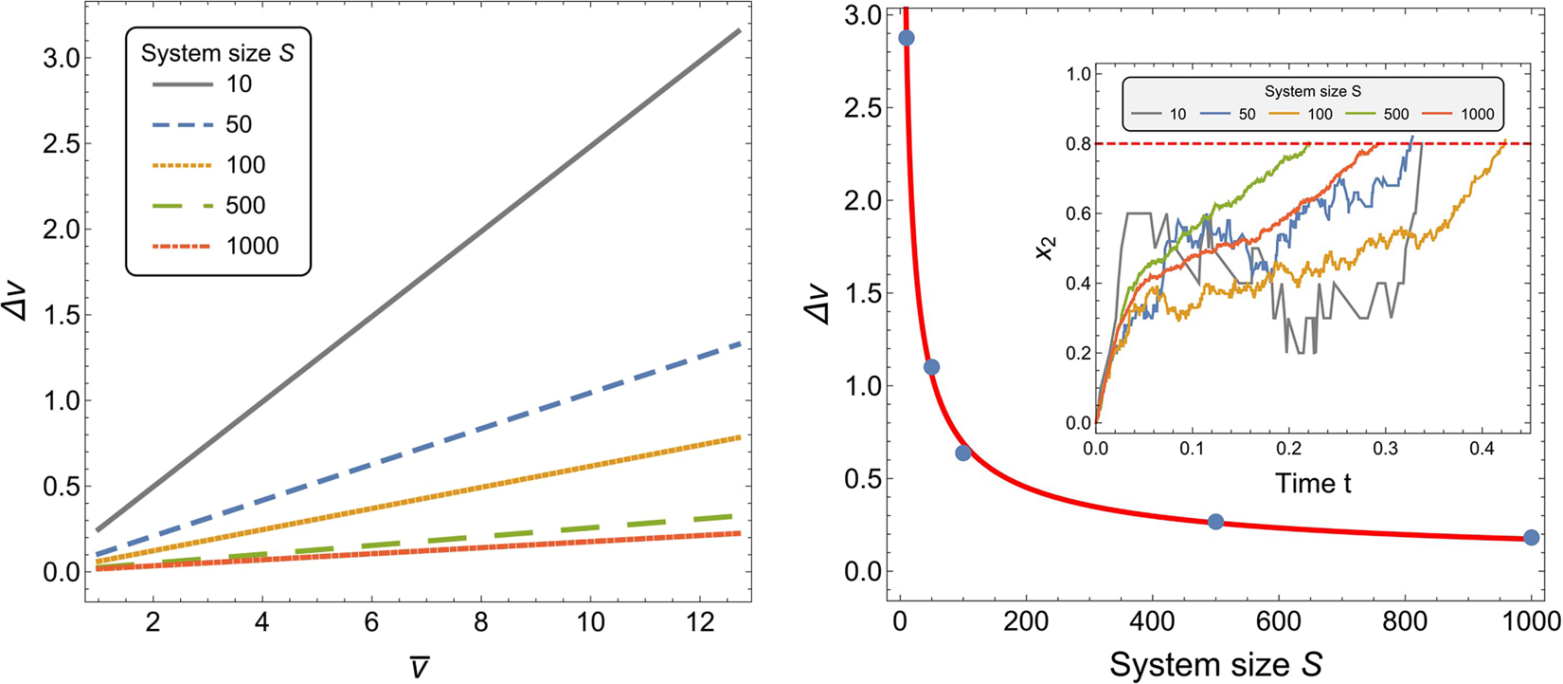
The just noticeable difference Δ*v* increases with the magnitude of random fluctuations. (left) the Weber fraction increases with decreasing system size *S* (*i*.*e*., for increasing random fluctuations), see Supplementary Table S1. (right) Just noticeable difference as a function of *S* for base quality $\bar{v}$. The data points are fitted with the red curve (*z* + *aS*^−*b*^), with *a* ≈ 12.13, *b* ≈ 0.626, *z* ≈ 0.013, and quality of fit *R*^2^ > 0.999 (see Sec. 5.4). The inset shows examples of commitment change for *v*_1_ = 10, *v*_2_ = 11 and varying number of bees *S*; the largest fluctuations are displayed for *S* = 10. Plots show results for signalling ratio *r* = 3.

As discussed by Thurstone^28^ and Stevens^3^, the discriminal variability has a key role in determining the just noticeable difference. The variability in correctly discriminating between two stimuli is directly proportional to the magnitude of the random fluctuations within the discrimination process. In the considered honeybee model, the magnitude of the random fluctuations is determined by the number of bees (*i*.*e*. the system size *S*): the smaller the system size, the larger the fluctuations. Figure 2 shows the effect of random fluctuations (by varying *S*) on the discriminability of the stimuli. In agreement with^3,28^, higher variability (*i*.*e*. higher fluctuations) leads to lower discriminability (*i*.*e*. higher just noticeable difference). This result contributes to the argument for the evolutionary advantages of group living by which collective decisions are more reliable than individual decisions^29,30^. Although, it is worth noting that in different systems accuracy is not always maximised through large group decisions^31,32^.

### Hick-Hyman’s Law

Hick-Hyman’s law^5,6^ states that the reaction time (RT) to a stimulus increases linearly with the amount of information *I* that needs to be processed:

4 $${\rm{RT}}=sI,$$

where *s* is the time taken by the organism to process one bit of information, and information *I* is a function of the number of alternatives *n* involved in the discrimination process.

Varying the task and its necessary computational resources varies the amount of processed information per alternative, ranging from a constant function (*i*.*e*. independent from *n*)^33^, to logarithmic^5,6^, to linear^34^, to exponential^35,36^.

**Figure 3:**
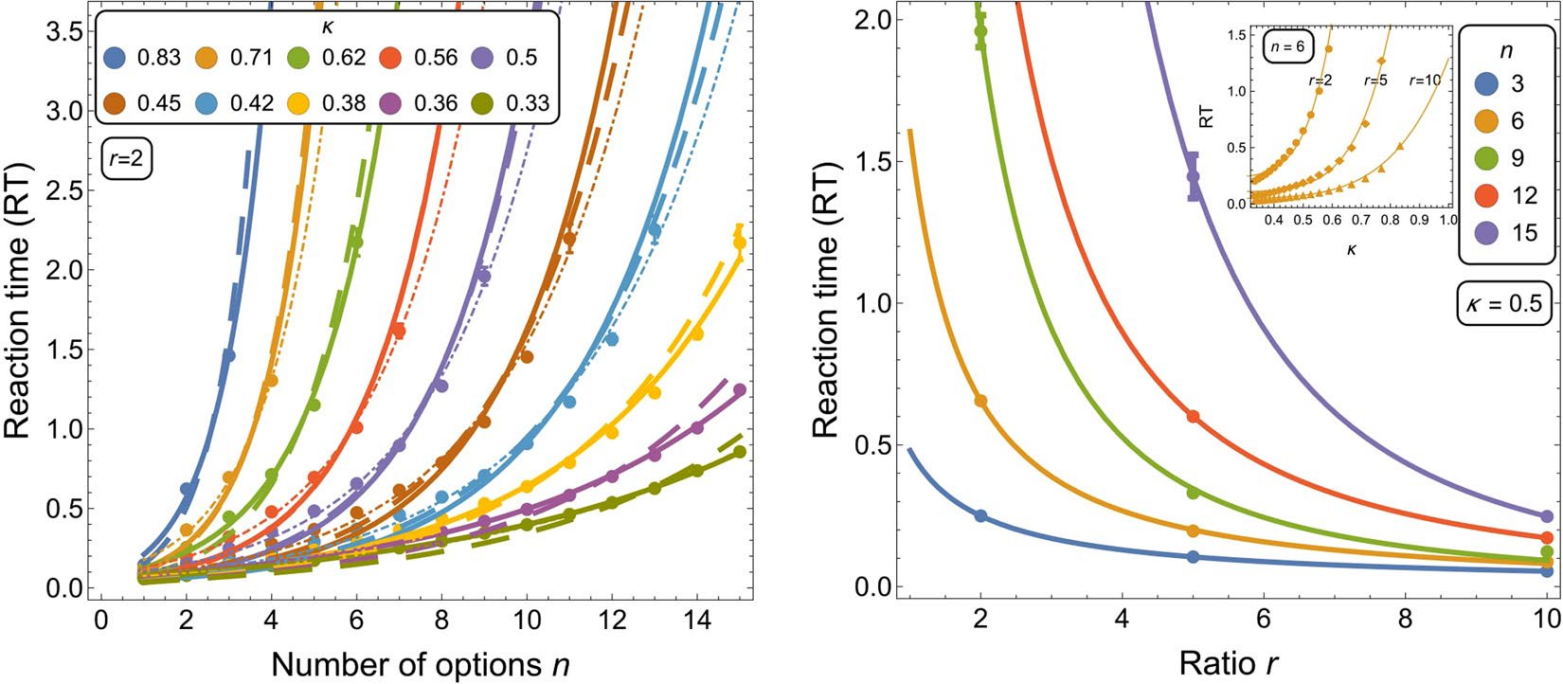
(Left panel) Mean RT to reach the quorum *Q* = 0.8 for the best option as a function of the number of options *n*. Different coloured lines correspond to varying decision difficulty *κ* (see Sec. 5.2). The SSA data (points) are fitted with curves of Eq. (5) (light dot-dashed lines), with curves of Eq. (7) (solid lines), and with curves of Eq. (7) using *f*_*μ*_(*κ*) (dashed thick lines) (see Supplementary Table S2). (Right panel) Mean RT decreases as a power law with the signalling ratio *r*. The SSA data (points) are fitted with the curve ${\rm{RT}}={z}_{2}{r}^{-{z}_{1}}$. The inset shows a power law increase (${\rm{RT}}={m}_{1}+{m}_{2}{\kappa }^{{m}_{3}}$) of RT as a function of the problem difficulty *κ*. Vertical bars show the variance.

The investigated task—value-sensitive best-of-*n* decision-making—is cognitively expensive as it requires the evaluation of *n* alternatives and the selection of the best-quality alternative if above acceptance threshold^18,37^. Neurological models applied to best-of-n decisions predict a non-linear increase of reaction time with the number of alternatives^38^. Considering the similarity between neurological and the collective decision-making models^39^, we expect a qualitative agreement with results on the same multi-alternative choice task. Indeed, Fig. 3 (left panel) shows an exponential increase of RT with the number of options *n* that is well approximated by the curve

5 $$I={e}^{{s}_{2}n},\quad {\rm{RT}}={s}_{1}{e}^{{s}_{2}n}$$

(see Sec. 5.2 for details). The nonlinear RT increase may possibly derive from nonlinearities of the process which are characteristic in decentralised systems^40^. This exponential increase in RT can be compensated for by increasing the signalling ratio *r* which produces a power law response (see Fig. 3, right panel). The trade-off between low *r* for discriminability (Fig. 1) and high *r* for speed resembles the well-known speed-accuracy trade-off and could be optimised through an increase of *r* over time (as proposed in^18^). The inset of Fig. 3 (right) shows that RT increases with increasing difficulty of the problem, however, in natural environments it is unlikely to find a large number of options with the equal high-quality value (*i*.*e*. *κ* ≈ 1). Additionally, during a decision, the honeybee colony may take a limited number of options into consideration, similarly to working memory in human brains^41^.

### Piéron’s Law

**Figure 4:**
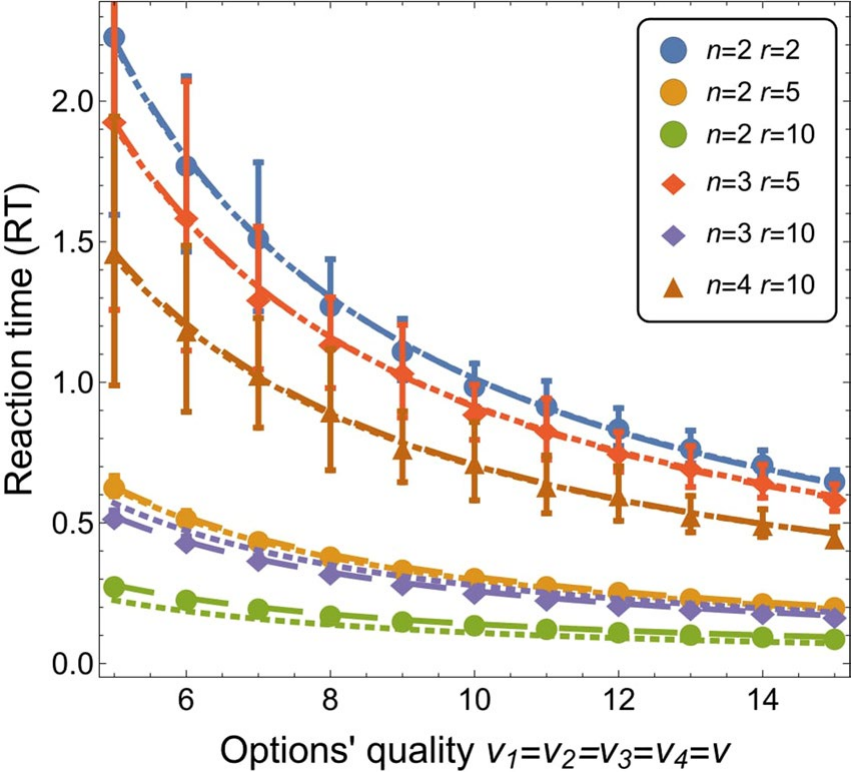
Mean RT (and variance) for decisions between *n* ∈ {2, 3, 4} equal-quality options as a function of the options’ quality *v* ∈ [5, 15] for varying values of the signalling ratio *r* ∈ {2, 5, 10}. The SSA data (points) are fitted with the power law curves of Eq. (6) (dashed lines) and with curves of Eq. (7) (dotted lines). See Supplementary Tables S3 and S4 for details on fitting values and relative quality.

Piéron’s law^7^ states that the mean RT decreases as a power law with increasing stimulus intensity *v*:

6 $${\rm{RT}}=a{v}^{-b},$$

where *a* and *b* are constants. (In the original formulation of the Piéron’s law the equation involves an additive constant term *z* as: *av*^−*b*^ + *z*, which we removed (*z* = 0).) The law has been initially proved to be valid on perception tasks in various sensory domains (such as pressure, temperature, taste of salt/sugar/bitter/acid, loudness, luminance)^7,8^. Later, studies have shown that also binary choice reaction tasks obey Piéron’s law^42–45^. Figure 4 shows that the considered model of value-sensitive decisions in a superorganism also obeys Piéron’s law.

We tested decision-deadlock breaking in equal-quality decisions so that we removed any possible overlapping effect from discriminability of stimuli and Weber’s law. As discussed in previous analysis^43,46^, the obtained agreement with Piéron’s law could be a natural artefact of optimality constraints in value-sensitive models. In the considered model (1), value-sensitivity is determined by value-dependent rates (Eq. (2)), which allowed previous analyses to approximate the honeybee model to simpler leaky competing accumulators and to prove its (quasi-)optimality^17,39^. Stafford and Gurney^43^ show that models composed of simple leaky integrators that accumulate evidence with speed proportional to the stimulus magnitude have reaction times that always fit Piéron’s law. Similarly, Van Maanen *et al*.^46^ show that optimal Bayesian classifiers which integrate evidence at a speed proportional to the input stimuli have reaction times that fit Piéron’s law. Our results support (and extend) these hypotheses by identifying a response in agreement with Piéron’s law on a model which has both positive and negative feedbacks (recruitment and cross-inhibition) proportional to the stimulus intensities.

### A Unifying RT law

Both Hick-Hyman and Piéron’s law s describe the variation in RT as a function of the decision problem, however, each law focuses on a different aspect. Here, we attempt to combine the two laws to identify a unique generic function that generalises Eqs (5) and (6) and determines the RT dynamics:

7 $${\rm{RT}}=\alpha {\bar{v}}^{-\beta }{e}^{\mu n},$$

where *α*, *β*, and *μ* are constants, and $\bar{v}$ is the mean quality of the *n* options. The parameter *μ* varies as a function of the decision problem difficulty, *i*.*e*. *μ* = *f*_*μ*_(*κ*). The function *f*_*μ*_ displays a power law increase with the problem difficulty *κ* in the form of ${f}_{\mu }(\kappa )={m}_{1}+{m}_{2}{\kappa }^{{m}_{3}}$, as shown in the inset of Fig. 3 (right panel). Eq. (7) reduces to Eq. (6) by imposing *a* = *αe*^*μn*^. The reduction to Eq. (5), however, is not straightforward because the term $\bar{v}$ is the mean option quality which varies with the number of options *n*, except in the special case of *κ* = 1. However the fitting results reported in Supplementary Table S2 show good agreement between parameters.

## Discussion

A large number of organisms at diverse levels of biological complexity, from humans to unicellular moulds, obey the same psychophysical laws that characterise the relationship between stimuli and the organism’s response. This study shows for the first time that groups of individuals, in our case honeybee colonies, considered as a single superorganism, might also be able to obey the same laws. Similarly to neurons, no individual explicitly encodes in its *simple* actions the dynamics determining the psychophysical laws; instead it is the group as a whole that displays such dynamics. The observed similarities in stimuli response between brain and superorganism motivate further investigations of collective behaviour through the lens of cognitive science and psychology^20–22,39,47,48^. Research synergies between neuroscience and collective intelligence studies can highlight analogies that could help better to understand both systems. Future work following this line of research aims at identifying which are the generic mechanisms causing psychophysical laws responses. Previous analyses showed how Weber’s law emerges from Bayesian collective decision-making^49^. In a similar fashion, other work has hypothesised that Piéron’s law responses are present in any optimal process with value-sensitive dynamics^43,46^. Further investigations on this topic may reveal which are the generic features that let a process exhibit psychophysical responses.

It is important to note that this study is a prediction of the honeybees behaviour based on the analysis of a computational model derived from field observations^20^, and the reported analysis holds only for the parameterisation of Eq. (2) (from^18^). Even if the investigated value-sensitive decision model^18^ is the state of art model with qualitative agreement with value-sensitive dynamics observed in honeybee swarms, our theoretical results still need empirical validation to confirm that superorganisms obey psychophysical laws. We are aware of one previous attempt to determine if superorganisms exhibit Weber’s law, through the study of house-hunting in the rock ant *Temnothorax albipennis*^50^; this study did not find Weber’s law. However the recruitment system of *Temnothorax*, while similar to that of honey bees, differs in some important regards. Rock ant colonies thus frequently split, especially when confronted with similar quality nest-sites, but are able to subsequently move to the best nest during a reunification phase^51^. This may relax selection pressure on decision-making mechanisms, which may thus diverge from displaying phenomena associated with optimal mechanisms.

An additional result of this study is the introduction of a novel psychophysical law that describes how the reaction time of value-sensitive decisions varies as a function of the number of options and the mean stimulus magnitude. The law is based on the combination of the Hick-Hyman’s and Piéron’s laws. While each law has been investigated independently and shown to hold for various stimuli, to the best of our knowledge the unified dynamics have not been taken into account in any study. The combined law predicts an organism’s response to variations in the number of options and in stimulus strength for value-sensitive decisions. Reaction time is determined by the interplay of these factors which are explicitly considered as function parameters in the combined law. We believe that the proposed formalisation is not limited to the analysis of collective behaviours but motivates empirical investigation by the psychology and neuroscience community. In fact, similarities between the considered honeybee model and neurological models in terms of their dynamics^38^ have been observed in this study and in previous work^39^.

## Methods

### Testing Weber’s law

To measure how the discriminability varies as a function of the stimuli strength, we followed the methodology of Deco *et al*.^4^. First, we collected simulation data from iterations of binary decision tasks in which we varied the base stimulus’s quality *v*_1_ ∈ [7.5, 12.5] with step size 0.5. For each base stimulus quality, we varied the second option’s quality *v*_2_ ∈ [*v*_1_ − 6, *v*_1_ + 6] with step size 0.1. We repeated the SSA for 100 independent runs for each pair of quality values for varying population size *S* ∈ {10, 50, 100, 500, 1000}. All simulations started from a fully uncommitted state, the individual behaviour coefficient (from Eq. (2)) was kept constant *k* = 1 and we varied the signalling coefficient *h* ∈ [1, 5].

**Figure 5:**
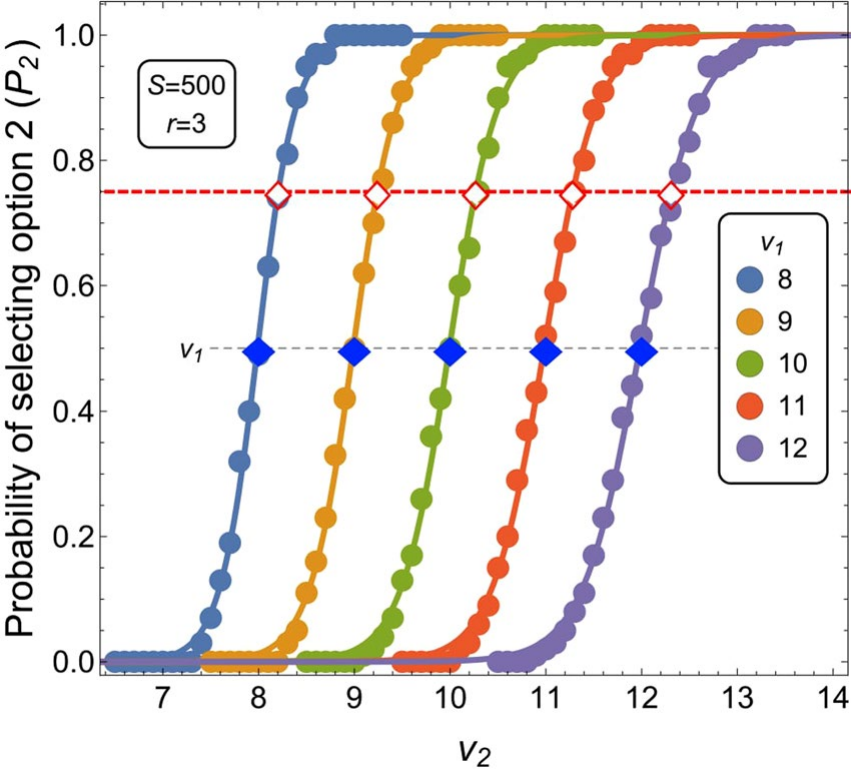
Proportion of runs with commitment to second option over quorum *Q* = 0.8 (circle points) for varying base stimulus quality *v*_1_ ∈ [8, 12] as a function of second option’s quality *v*_2_ (on the x-axis). SSA data are fitted to the curve ${P}_{2}={\mathrm{(1}+{e}^{b(a-{v}_{2})})}^{-1}$. The red hollow diamonds indicate the intersection of the fitted curves with discrimination in favour of option 2 in 75% of the cases, while the blue filled diamonds highlights the *v*_1_ values.

We considered that an option was selected when the quorum *Q* = 0.8 of the population (*i*.*e*., 80% of *S*) became committed to that option. For each base option’s quality *v*_1_, we computed the probability *P*_2_ of selecting the second option as a function of its quality *v*_2_ (see Fig. 5). We fitted the data with a logistic function ${P}_{2}={\mathrm{(1}+{e}^{b(a-{v}_{2})})}^{-1}$ with *a* and *b* fitted parameters. Through the fitted curve, we computed the second option’s value ${v}_{2}^{\ast }$ for *P*_2_ = 0.75 (red hollow diamonds in Fig. 5). The difference between ${v}_{2}^{\ast }$ and *v*_1_ determined the just noticeable difference Δ*v* for the mean quality $\bar{v}=({v}_{1}+{v}_{2}\mathrm{)/2}$. Finally, we fitted the just noticeable difference values through a line ${\rm{\Delta }}v=w\bar{v}$ to determine the Weber fraction *w*. Supplementary Table S1 shows the fitted values and the quality of fit *R*^2^ (for details on *R*^2^ see Sec. 5.4).

### Testing Hick-Hyman’s law

To investigate how the reaction time (RT) varies as a function of number of options *n*, we considered the best-of-*n* case in which there is one superior option and *n* − 1 inferior quality distractors, in agreement with the experimental setup of empirical studies^18,52–54^. We fixed the quality of the inferior *n* − 1 distractors to *v*_*d*_ = 5 and varied the quality of the superior option 1, *v*_1_ ∈ [5, 15] with step size 0.5. The problem difficulty is determined by the ratio between the distractors’ quality and the best option’s quality: *κ* = *v*_*d*_/*v*_1_. Simulations started from a fully uncommitted state and stopped when commitment to any option reached the quorum *Q* = 0.8, or the maximum time *T* = 100 was reached. The number of options varied as *n* ∈ [1, 15], system size was *S* = 1000, individual behaviour coefficient was a constant *k* = 1, and the signalling coefficient varied as *h* ∈ {2, 5, 10}. We ran 100 independent runs for each parameterisation. The case *n* = 1 represents the base reaction time to a stimulus.

Figure 3 shows how the mean RT varies as a function of the considered parameters, and Supplementary Table S2 shows the fitted curves’ parameters.

### Testing Piéron’s law

To investigate the agreement with the Piéron’s law, we considered decisions with equal-quality options. The system needs to break the decision deadlock and select any option (with committment over quorum *Q* = 0.8). We ran 100 independent runs for *S* = 1000, *k* = 1, *h* ∈ {2, 5, 10} and options’ quality *v* ∈ [5, 15]. Supplementary Table S3 shows the fitting data for the curves displayed in Fig. 4.

### Quality of fit

We measured the quality of fit with the coefficient of determination, denoted *R*^2^, which is the proportion of the variance in the dependent variable that is predictable from the independent variables. Better fits correspond to higher *R*^2^ ∈ [0, 1].

### Open source data

Simulation code (in python) and scripts (in bash) to generate the data, the generated data, and *Wolfram Mathematica* notebooks to analyse data are open source and available online at: http://diode.group.shef.ac.uk/extra_resources/Data_Reina_PsychoLaws.zip.

## Electronic supplementary material

Supplementary Information
