# Supplementary material for: Psychophysical Laws and the Superorganism
Source: Sci Rep. 2018 Mar 12;8:4387. doi: 10.1038/s41598-018-22616-y (PMC5847525; doi:10.1038/s41598-018-22616-y)
Supplement: Supplementary file 1 — Supplementary Information [file 41598_2018_22616_MOESM1_ESM.pdf]

SUPPLEMENTARY INFORMATION

Psychophysical Laws and the Superorganism

Andreagiovanni Reina[1,*], Thomas Bose[1], Vito Trianni[2], and James A. R. Marshall[1]

[1]Department of Computer Science, University of Sheffield, S1 4DP, UK
[2]ISTC, Italian National Research Council, Rome, Italy

[*]Corresponding author: a.reina@sheffield.ac.uk

Supplementary Table S1: Fitted parameters for the line $\Delta v = w\bar{v}$ for varying system size S and signalling ratio $r = h/k$. Value R^2 indicates the quality of fit.

S	r	w	R^2
10	1	0.211	0.9999
10	2	0.229	0.9999
10	3	0.248	0.9998
10	4	0.259	0.9997
10	5	0.272	>0.9999
50	1	0.064	0.9988
50	2	0.083	0.9998
50	3	0.105	0.9999
50	4	0.118	>0.9999
50	5	0.125	0.9999
100	1	0.038	0.9997
100	2	0.051	0.9997
100	3	0.062	0.9999
100	4	0.071	>0.9999
100	5	0.082	>0.9999
500	1	0.018	0.9996
500	2	0.023	0.9994
500	3	0.026	0.9997
500	4	0.03	0.9998
500	5	0.034	0.9998
1000	1	0.013	0.9988
1000	2	0.015	0.9994
1000	3	0.018	0.9993
1000	4	0.019	0.9994
1000	5	0.02	0.9991

Supplementary Table S2: Fitted parameters for the curve of Eq. (5), RT = $s_1 e^{s_2 n}$ (see Fig. 3(left)) for varying signalling ratio $r \in \{2, 5, 10\}$ and decision difficulties κ. Value E indicates the quality of fit.

r	κ	s_1	s_2	R^2	α	β	μ	R^2
2	1.	0.014	2.541	>0.9999	0.085	1.121	2.527	0.9998
2	0.83	0.084	0.954	0.9967	0.49	0.981	0.914	0.9973
2	0.71	0.091	0.668	0.9984	0.055	0.01	0.819	0.9969
2	0.62	0.074	0.562	0.9985	0.075	0.01	0.561	0.9985
2	0.56	0.084	0.42	0.9991	0.049	0.01	0.515	0.9941
2	0.5	0.075	0.361	0.9984	0.041	0.01	0.442	0.9868
2	0.45	0.064	0.318	0.9969	0.034	0.01	0.389	0.9836
2	0.42	0.061	0.274	0.997	0.036	0.02	0.326	0.9873
2	0.38	0.061	0.235	0.9976	0.063	0.012	0.234	0.9976
2	0.36	0.078	0.184	0.9993	0.203	0.482	0.175	0.9995
2	0.33	0.078	0.161	0.9988	0.4	0.798	0.143	0.9999
5	1.	0.059	1.162	0.9991	0.364	1.062	1.123	0.999
5	0.83	0.066	0.601	0.999	0.49	1.155	0.581	0.9991
5	0.71	0.06	0.426	0.9979	0.061	0.01	0.425	0.9979
5	0.62	0.03	0.407	0.9901	0.032	0.01	0.402	0.9893
5	0.56	0.012	0.406	0.9806	0.478	1.923	0.364	0.9738
5	0.5	0.035	0.243	0.993	0.037	0.016	0.242	0.9929
5	0.45	0.055	0.163	0.9994	0.115	0.39	0.157	0.9995
5	0.42	0.054	0.138	0.9991	0.202	0.674	0.125	0.9999
5	0.38	0.05	0.122	0.9985	0.209	0.709	0.106	0.9999
5	0.36	0.045	0.113	0.9984	0.17	0.639	0.096	0.9998
5	0.33	0.041	0.105	0.9982	0.146	0.59	0.088	0.9997
10	1.	0.033	0.948	0.9977	0.187	1.037	0.929	0.9968
10	0.83	0.044	0.428	0.9962	0.045	0.01	0.428	0.9962
10	0.71	0.017	0.394	0.9827	0.172	1.33	0.381	0.9803
10	0.62	0.006	0.377	0.9691	0.016	0.296	0.333	0.9617
10	0.56	0.042	0.15	0.9992	0.089	0.407	0.146	0.9993
10	0.5	0.04	0.122	0.9991	0.151	0.699	0.11	0.9999
10	0.45	0.036	0.105	0.9986	0.145	0.703	0.091	0.9999
10	0.42	0.033	0.095	0.9985	0.108	0.594	0.08	0.9998
10	0.38	0.029	0.088	0.9987	0.078	0.472	0.075	0.9998
10	0.36	0.026	0.082	0.9989	0.059	0.376	0.071	0.9998
10	0.33	0.024	0.077	0.9991	0.048	0.31	0.067	0.9998

Supplementary Table S3: Fitted parameters for the curve RT $= av^{-b}$ (see Fig. 4) for varying signalling ratio r. Value R^2 indicates the quality of fit.

r	n	a	b	R^2	α	β	μ	R^2
2	2	13.675	1.13	0.9998	0.085	1.121	2.527	0.9998
3	2	6.291	1.035	0.9999	0.828	1.035	1.013	0.9999
3	3	62.303	1.13	0.9993	0.828	1.035	1.013	0.9999
4	2	4.641	1.059	0.9999	0.287	1.06	1.392	0.9997
4	3	18.654	1.059	0.9998	0.287	1.06	1.392	0.9997
5	2	3.289	1.028	>0.9999	0.364	1.062	1.123	0.999
5	3	10.906	1.078	0.9998	0.364	1.062	1.123	0.999
6	2	2.532	1.014	0.9999	0.4	1.052	0.96	0.9995
6	3	7.308	1.063	0.9995	0.4	1.052	0.96	0.9995
7	2	2.039	0.991	>0.9999	0.412	0.998	0.807	0.9999
7	3	4.645	0.999	0.9999	0.412	0.998	0.807	0.9999
7	4	28.809	0.841	0.9983	0.412	0.998	0.807	0.9999
8	2	1.839	1.007	>0.9999	0.083	0.806	1.164	0.9799
8	3	3.706	1.001	0.9999	0.083	0.806	1.164	0.9799
8	4	24.017	1.152	0.999	0.083	0.806	1.164	0.9799
9	2	1.593	1.006	>0.9999	0.121	0.959	1.073	0.9922
9	3	3.184	1.031	0.9999	0.121	0.959	1.073	0.9922
9	4	13.525	1.133	0.9988	0.121	0.959	1.073	0.9922
10	2	1.372	0.989	>0.9999	0.187	1.037	0.929	0.9968
10	3	2.699	1.02	>0.9999	0.187	1.037	0.929	0.9968
10	4	7.9	1.048	0.9998	0.187	1.037	0.929	0.9968
15	2	0.933	1.019	>0.9999	0.043	0.892	1.015	0.9811
15	3	1.48	1.006	>0.9999	0.043	0.892	1.015	0.9811
15	4	2.528	0.974	0.9997	0.043	0.892	1.015	0.9811
15	5	9.646	1.019	0.9986	0.043	0.892	1.015	0.9811
20	2	0.642	0.997	>0.9999	0.197	1.023	0.554	0.9982
20	3	1.007	0.996	>0.9999	0.197	1.023	0.554	0.9982
20	4	1.718	1.028	0.9999	0.197	1.023	0.554	0.9982
20	5	3.202	1.026	0.9994	0.197	1.023	0.554	0.9982

Supplementary Table S4: Fitted parameters for the curve RT $= \alpha v^{-\beta} e^{f_\mu(\kappa)n}$ with $f_\mu(\kappa) = m_1 + m_2\kappa^{m_3}$ for varying signalling ratio r. Value R^2 indicates the quality of fit.

r	α	β	m_1	m_2	m_3	R^2
2	0.132	0.555	0.027	1.619	2.062	0.9845
5	0.112	0.624	0.063	1.11	2.696	0.977
10	0.104	0.891	0.097	0.896	3.671	0.9695
